# Exploring Biomarkers to Predict Thrombogenic Risk in Pregnancy

**DOI:** 10.3390/jcm14030932

**Published:** 2025-01-31

**Authors:** Catalina Filip, Daniela Roxana Matasariu, Alexandra Ursache, Cristina Daniela Dimitriu, Cristiana Filip, Vasile Lucian Boiculese, Demetra Gabriela Socolov

**Affiliations:** 1 Department of Vascular Surgery, University of Medicine and Pharmacy “Grigore T. Popa”, 700111 Iasi, Romania; catalina.filip@umfiasi.ro; 2 Department of Vascular Surgery, CHU “Gabriel Montpied”, 63000 Clermont-Ferrand, France; 3 Department of Obstetrics and Gynecology, University of Medicine and Pharmacy “Grigore T. Popa”, 700111 Iasi, Romania; alexandra.ursache@umfiasi.ro (A.U.); demetra.socolov@umfiasi.ro (D.G.S.); 4 Department of Obstetrics and Gynecology, Cuza Voda Hospital, 700038 Iasi, Romania; 5 Department of Morpho-Functional Sciences II, University of Medicine and Pharmacy “Grigore T. Popa”, 700111 Iasi, Romania; cristina.dimitriu@umfiasi.ro; 6 Department of Biochemistry, University of Medicine and Pharmacy “Grigore T. Popa”, 700111 Iasi, Romania; cristiana.filip@umfiasi.ro; 7 Biostatistics, Department of Preventive Medicine and Interdisciplinarity, University of Medicine and Pharmacy “Grigore T. Popa”, 700111 Iasi, Romania; lboiculese@gmail.com

**Keywords:** thrombosis, coagulation biomarkers, pregnancy, thrombophilia, protein C, protein S, antiphospholipid antibodies, human factor V, beta-2-glycoprotein

## Abstract

**Background:** Normal pregnancy and the postpartum period are characterized by thrombotic predisposition. Consequently, monitoring coagulation markers and conducting risk assessments are essential in preventing thrombotic events that negatively impact both the mother and the child. In medical practice, fibrinogen/fibrin degradation products (FDPs) are the main coagulation markers currently investigated in pregnancy. **Methods:** We investigated proteins C and S, antiphospholipid antibodies (APLs), human factor V, and beta-2-glycoprotein 1 or apolipoprotein H (APOH) levels to determine whether there is any difference between normal third-trimester pregnancies and pregnant women in late pregnancy who end up developing deep vein thrombosis (DVT). **Results:** Our data show a significant correlation between protein C levels and the number of weeks of pregnancy, as well as statistically significant differences between healthy pregnant women and pregnant women with DVT in terms of the values of FDP, protein S, and APL. The DVT group had higher FDP levels but lower AFL and protein S values. **Conclusions:** Given the significant prothrombotic correlation that exists between proteins C and S, we propose that variations in their levels can serve as valuable markers in the evaluation of thrombotic risk during the final stages of pregnancy.

## 1. Introduction

Pregnancy, as well as the postpartum period, is characterized by a hypercoagulable status that prevents excessive blood loss [1]. In normal pregnancy, mild inconveniences such as leg swelling or moderate thrombotic events (e.g., phlebitis) can emerge [2]. However, severe thrombotic events such as venous thromboembolism (VTE) or pulmonary embolism (PE) may occur, particularly during the third trimester of pregnancy or within the first six weeks postpartum [2].

The literature indicates that, in developed countries, thromboembolic complications such as acute pulmonary embolism are the leading causes of perinatal mortality [2,3,4,5]. The thrombotic risk significantly increases when pregnancy is associated with predisposing conditions such as a personal history of thrombosis, antiphospholipid syndrome, lupus, preeclampsia, hereditary or acquired thrombophilia, and, more recently, COVID-19 infection [1,2]. The postpartum period presents an even higher thrombotic risk, up to ten times greater than the antepartum timeframe [2].

Other risk factors, including obesity, smoking, immobility, myeloproliferative neoplasms, JAK2 (Janus Kinase 2) mutation, multiple pregnancies, and advanced maternal age, increase the thrombogenic risk by 1.5–2 times [6,7,8,9,10]. Assisted reproduction techniques have been shown to elevate the thrombogenic risk by as much as tenfold [11]. Additionally, in pregnancies associated with hereditary thrombophilia, the VTE risk is up to 30 times higher than in pregnancies without such conditions [12].

During pregnancy, an increased thrombogenic risk negatively impacts both maternal and fetal outcomes [1,10]. Therefore, assessing specific markers to detect inherited risk factors or deficiencies in blood clotting is particularly important, especially in asymptomatic patients [11]. This research aims to investigate the physiological changes in certain coagulation and thrombosis-related markers during pregnancy. By determining their baseline levels, we hope to improve the understanding of VTE physiopathology, enhance risk stratification, and support more accurate VTE diagnosis.

Among the key biomarkers implicated in thrombosis are PDF, proteins C and S, antiphospholipid antibodies (APLs), factor V Leiden, and βeta-2-glycoprotein 1 (APOH) [11,12]. A specific fraction of FDP is represented by the D-dimer, a biomarker used as a criterion for evaluating the risk of venous thrombosis [13]. Due to the fact that D-dimer evaluation is not widely available in general hospitals and the evaluation of FDP offers a more accessible and faster opportunity to assess thrombotic risk [12,13], we selected it for our investigation. FDPs are fragments of proteins that are produced when fibrin, also a protein involved in blood clotting, is broken down by the enzyme plasmin. These fragments are markers of fibrinolysis, the process which dissolves blood clots. FDPs include smaller fragments such as the D-dimer, as well as other fibrin and fibrinogen degradation components [13,14,15]. Proteins C and S are key components in blood coagulation control, being involved in the anticoagulant pathway [16]. Circulating protein C is the zymogen form of an anticoagulant serine protease, activated protein C (APC). The activation occurs on the endothelial cell surfaces through the combined action of the thrombin–thrombomodulin complex and the endothelial protein C receptor (EPCR). Once activated, APC degrades the regulatory proteins of coagulation factors VIII-a and V-a in the presence of its cofactor, protein S. Deficiencies in protein C or S are strongly associated with the development of deep vein thrombosis (DVT) [8,9,16]. Recent findings suggest that protein S not only acts as a cofactor for APC but also exhibits a direct anticoagulant effect by modulating both the initiation and propagation phases of coagulation [16]. Furthermore, APC activity is significantly diminished in the absence of protein S. The anticoagulant properties of protein S are thought to depend on its ability to bind efficiently to negatively charged phospholipid surfaces, underscoring its critical role in maintaining hemostasis. Deficiencies in either protein can therefore predispose individuals to a prothrombotic state [16].

Antiphospholipid syndrome (APS) is a systemic autoimmune disorder characterized by venous or arterial thrombosis. Cellular membranes contain phospholipid (PL) molecules that, when disrupted by viral or bacterial infections, release fragments into the bloodstream. These fragments can stimulate the generation of antibodies that target both phospholipids and phospholipid-binding proteins. Among the phospholipid-binding proteins with anticoagulant functions are proteins C and S. The major antiphospholipid (APL) antibodies include lupus anticoagulant (LA), anticardiolipin antibodies (aCLs), and anti-beta-2-glycoprotein 1 antibodies or apolipoprotein H (APOH). In pregnancy, the presence of antiphospholipid antibodies can lead to severe complications ranging from thrombosis to fetal growth restriction or even fetal loss [11,17,18,19]. APS can remain silent in long-term asymptomatic APL-positive patients, but, during pregnancy, silent APS may cause unfavorable outcomes. APOH, also known as beta 2 glycoprotein 1, is a multifunctional plasma protein with key roles in both physiological and pathological processes, particularly in hemostasis and immunity, containing the main antigenic target of autoantibodies associated with APS [20,21]. Studies indicate that APOH, along with protein C and thrombomodulin, plays a dual role, either promoting or inhibiting inflammatory and coagulation responses depending on external signals. Dysfunctions in membrane structure and the proteins involved in coagulation, such as protein C, protein S, and APOH, contribute to thrombogenic events [21]. Factor V is another key player in both procoagulant and anticoagulant processes. Its genetic mutations cause hereditary thrombophilia to be associated with DVT, but the exact thrombotic mechanism remains to be revealed [11].

The evaluation of these proteins, especially during pregnancy, is essential due to their critical roles in coagulation and the risk of thrombophilia. While PDF or D-dimer testing is commonly performed in pregnancy when clinical suspicions arise, determining protein C, protein S, APL, human factor V, and APOH levels is not routinely conducted. Given the heightened thrombogenic risk and the potential for silent thrombophilia during pregnancy, a comprehensive assessment of these biomarkers is crucial. This study focuses on evaluating these coagulation biomarkers in the third trimester of normal pregnancies and those with thrombotic events.

## 2. Materials and Methods

This study included patients hospitalized at the obstetrics and gynecology “Cuza Voda” Maternity Hospital, Iasi, Romania, between September 2021 and September 2023. This study was conducted in accordance with the Declaration of Helsinki and approved by the Ethics Committee of the University of Medicine and Pharmacy “Grigore T. Popa”, Iasi (105/22 July 2021), and the Obstetrics and Gynecology Hospital “Cuza-Voda”, Iasi, Romania (10426/24 August 2021). Informed consent was obtained from all the subjects involved in this study.

We included pregnant women between 23 and 42 weeks of gestation. We specifically chose this timeframe because severe thrombotic events occur with a higher frequency in this period. We excluded all patients with confirmed acquired or inherited thrombophilia. We also excluded all pregnant women with autoimmune diseases or any other treatment that may have altered the results of serum thrombotic-related markers.

We divided the included pregnant women into two groups. The control group comprised healthy women without any other associated pathology or concomitant risk factors, such as gestational diabetes, hypertension, preeclampsia, systemic lupus erythematosus, hereditary or acquired thrombophilia, liver disease, renal pathology, multiple pregnancies, inferior limb varicose veins associated with venous insufficiency, and a previous thrombotic episode, and with no medication that could alter the coagulation profile. The second group enrolled singleton pregnant women who had experienced a thrombotic event during pregnancy but were negative for acquired or inherited thrombophilia, negative for the abovementioned risk factors, and also without any medication that could alter the coagulation and fibrinolytic system. All included pregnant women were in their first or second pregnancy, and we excluded multiparous women as a possible risk factor for DVT.

The serum samples were analyzed, and the abovementioned thrombotic markers (D-dimer APL, proteins C and S, and APOH) were dosed. For serological determination, standardized ELISA kits for research-only purposes were used for the following fibrinogen/fibrin degradation products (FDPs): APL, APOH, and human protein C (PROC) from My BioSource, human factor V (factor V) from AssayPro, and human protein S (PROS1) from Novus Biologicals.

### Statistical Analysis

The data were collected and initially preprocessed in Microsoft Excel. We checked the correctness of the values through the range of variation. We used SPSS 24 for the statistical analyses (IBM Corp. Released 2016. IBM SPSS Statistics for Windows, Version 24.0. Armonk, NY, USA: IBM Corp.).

To describe the data, the following measures were calculated: sample size, absolute and relative frequencies, mean, standard deviation, confidence interval (95%) of the mean, quartiles, minimum, and maximum.

For the statistical analysis, we used the non-parametric Mann–Whitney hypothesis tests aimed at real continuous variables and the Chi-Square and Fisher tests for frequency comparisons. The critical point for significance was *p* = 0.05.

To study the possible relationships between the data, we applied simple linear regression and the multivariate logistic method. We determined the correlations and coefficients of the regression models and described the effects found.

## 3. Results

After applying the inclusion and exclusion criteria, we obtained a total of 43 healthy pregnant controls and 29 pregnant women with thrombosis during pregnancy.

**Figure 1:**
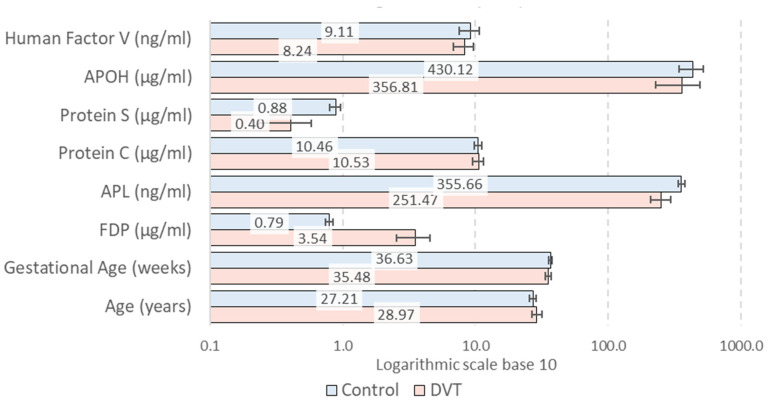
The averages and confidence intervals (95% precision) for clinical parameters of DVT vs. control. (APOH-apolipoprotein H; APL-antiphospfolipid; FDP-fibrinogen/fibrin degradation products; DVT-deep vein thrombosis).

The control group consisted of pregnant women aged 18 to 37 years, all in their third trimester, between 28 and 42 weeks of gestation. The thrombosis group encompassed pregnant women aged between 18 and 41, with singleton pregnancies ranging from 23 to 41 weeks of gestation. Figure 1 illustrates the average age and duration of pregnancy for both groups.

The statistics of the demographical data and their coagulation parameter patterns are depicted in Table 1.

**Figure 2:**
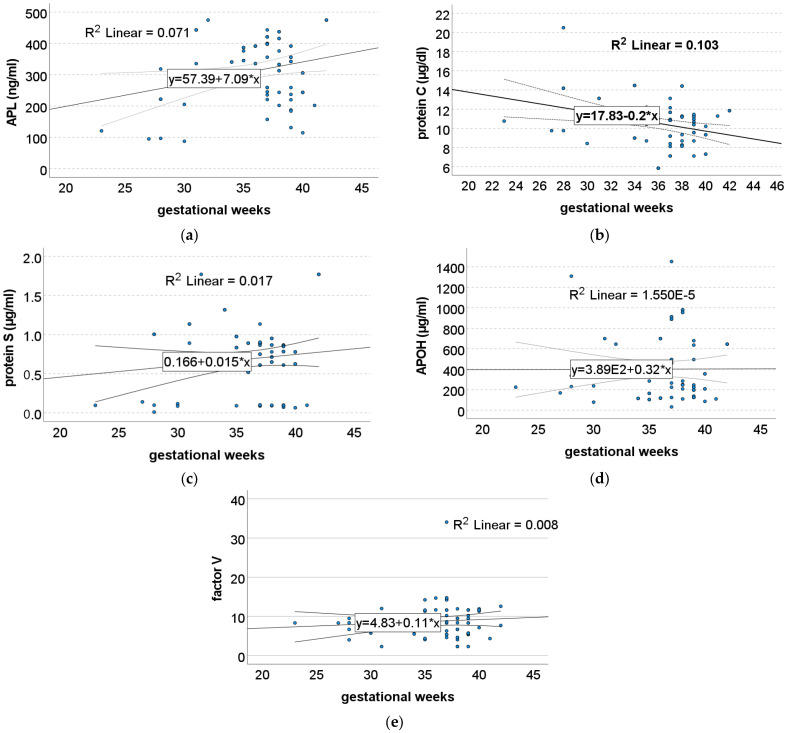
Simple linear regression throughout gestation for (**a**) APL, (**b**) protein C, (**c**) protein S, (**d**) APOH, and (**e**) human factor V.

While there were no notable differences between our two study groups concerning the demographic characteristics, women’s age, and gestational duration, we detected distinct coagulation patterns with statistically significant results. The obtained data revealed significant differences for three of our included variables, as indicated by the Mann–Whitney U test. The FDP levels were significantly higher in the DVT group compared to the controls, with a *p*-value of less than 0.001 (Table 2). Moreover, the APL and protein S levels were significantly lower in the DVT group compared to the control group, with a *p*-value < 0.001 (Figure 2a,c). No statistically significant patterns were observed for the remainder of the evaluated coagulation markers.

Another interesting observation we made was that the protein C serum levels seemed to decrease linearly depending on the gestational age of the pregnancy (Figure 2b). The value of protein C decreased by about 0.203 per week of gestation, being negatively correlated with the gestational age of the pregnancy. In reverse, the APL seemed to be significantly directly correlated with the gestational age, with a 7.087 increase per week of gestation (0.266 Pearson’s correlation) (Figure 2a). The correlations between the protein S value and the gestational age, as well as the APOH and factor V correlations, were not statistically significant, with a weak direct correlation. The three values increased by 0.015 per week of gestation (Figure 2c), 0.325 per week of gestation (0.004 Pearson’s correlation) (Figure 2d), and 0.109 per week of gestation, respectively (0.091 Pearson’s correlation) (Figure 2e).

When we performed a multivariate statistical analysis, applying multiple logistic regressions, we only detected one significant co-variable: protein S. If the protein S value decreased to 0.1, there was a 1.48 increased OR of suffering from DVT.

## 4. Discussion

This study aimed to evaluate whether, in addition to well-established risk factors for thrombosis such as hypertension, diabetes, multiparity, obesity, varicose veins, and personal history of thrombosis, there are alterations in the coagulation markers in pregnant women who develop thrombotic events during pregnancy compared to healthy pregnant controls. Furthermore, we hope our results will help develop a risk stratification scale based on the combined assessment of the clinical risk factors and a minimal evaluation of the most-impacting serum coagulation markers.

The obtained FDP values for both our groups align with those reported in the literature, which indicate an interval of 9.32–12.83 μg/mL for the third trimester of pregnancy [22]. However, the data in the literature show significant variation, possibly due to population differences. A value of 7.7 μg/mL was proposed by Wada et al. in 2008 as a cutoff negative predictive value for DVT [23]. Wang et al. stated that the FDP value increases throughout pregnancy, with a 0.15–7.40 range in the second trimester and a 0.55–13.43 range in the final trimester [24]. Given this variability in PDF levels and their lack of specificity and sensitivity in detecting DVT, although the difference between our two groups was statistically significant, we considered it necessary to explore additional coagulation biomarkers, including protein C, protein S, APL, APOH, and factor V, for a more pertinent conclusion.

Regarding APL levels in the last trimester of normal pregnancy, there is a notable scarcity of data in the literature. Moreover, the serological determination of APL is complex due to several factors, including patient heterogeneity, the nature of the antigenic targets, and variability in the cutoff values used to define positivity [25,26]. Nevertheless, it is generally accepted that higher antibody titers, particularly those involving APOH, are associated with a more severe disease course, reflected by an increased risk of venous thrombosis [25,26], pulmonary embolism [27], and pregnancy loss [28,29]. The kit employed in our study detects all antiphospholipid antibodies present in the patient’s serum, regardless of their immunoglobulin (Ig) M, Ig G, or Ig A isotype. Consequently, specific antibodies for various phospholipid antigens were identified, and we believe our data can be applicable in assessing thrombogenic risk during late pregnancy. However, we detected lower levels of APL in the DVT group, suggesting a non-prothrombotic state, thus underlining the fact that this form of evaluation is not useful in assessing thrombotic risk in healthy pregnant women.

Our data regarding circulating APOH levels are not consistent with the ranges reported in the literature, presenting a wide variability in both the DVT and the control group. Studies suggest that circulating human β2-glycoprotein 1 levels range between 150–300 μg/mL [30,31,32] and 50–500 μg/mL [33]. Our control group presented almost twice the value reported in the literature, ranging from almost 104 to 980 μg/mL. The values from the DVT group also varied from 32 to 1452 μg/mL, with only one case having an APOH value of under 50 μg/mL. β2-glycoprotein 1, also known as APOH, is a membrane-adhesion glycoprotein with multifunctional roles, including binding to cardiolipin, which enhances its anticoagulant effect [34,35,36]. The literature indicates that APOH levels are reduced in pregnant women, while, paradoxically, higher levels have been observed in patients with antiphospholipid syndrome [36,37]. All of our included pregnant women tested negative for antiphospholipid syndrome. We believe that this discrepancy is generated by the fact that all of our included participants were in late pregnancy, at more than 23 weeks of gestation. It is generally accepted that APOH deficiency could precipitate a prothrombotic event in a thrombogenic state [29,38]. Our findings align with other results from the literature suggesting that APOH assessment lacks substantial clinical utility in the evaluation of thromboembolic events during advanced normal pregnancy [39].

The serum protein C concentrations observed in our study were higher than those reported in the literature. The normal range is generally cited to be approximately 4.8 μg/mL for both non-pregnant and pregnant individuals [40,41]. We also attributed this discrepancy to the fact that our data specifically pertained to the last trimester of pregnancy, whereas the literature does not specify the gestational period under investigation. Additionally, we observed an indirect statistical correlation between the gestational week of pregnancy and the protein C levels, which we consider important in assessing thrombotic risk at the end of pregnancy, a correlation consistent with data found in the literature [42].

Regarding protein S, most previous studies provide data on its concentrations in healthy adults, which range from 16 to 25 μg/mL [17,43,44,45]. However, limited data exist regarding protein S levels during pregnancy, particularly in the third trimester. Our findings for protein S concentrations during late pregnancy are much lower than those reported for non-pregnant adults [43]. The kit we used indicates a normal range of 16.8 μg/mL for healthy adults, with a minimum detectable concentration of 0.23 μg/mL. The values obtained in our study fall within the kit’s range of accurate detection, and we consider them to be specific for late pregnancy [42,46]. Literature reports on protein S levels during pregnancy are inconsistent, with some suggesting that protein S levels remain unchanged throughout pregnancy, while others indicate a decline between the first and second trimesters, with no further reduction in the third trimester [47]. We detected a statistically significant lower level of protein S in the DVT group compared to the controls and a linear decrease directly proportional to the gestational age during pregnancy, thus suggesting that evaluation in late pregnancy might help identify pregnant women at risk of developing thrombotic events [48].

As a key participant in the coagulation process, factor V must undergo a proteolysis phase in order to achieve activation and contribute to clot formation, but it also exhibits an anticoagulant effect mediated by APC [49]. The values we obtained fall into the normal range of the kit we used to perform our evaluations. In the literature, there are scarce data regarding the serum evaluation of factor V, and most of the research focuses on evaluating its activity [50,51]. The only normal range values that we found were published by Tracy et al. and were situated between 4 and 14 μg/mL [50]. The results obtained when assessing the serum value in the pregnant women with DVT in our study align with the normal range cited in the literature, but the control group values are above the normal interval, perhaps indicating a DVT-protective effect of higher factor V plasma levels. The value did not reach statistical significance, but we believe that it needs further evaluation on larger cohorts to strengthen our results and fully understand its role.

Finally, our findings should be interpreted in light of the study’s limitations. Firstly, the sample size was small, limiting the generalizability of our results to a broader population. The small cohort size was the result of the restrictive inclusion and exclusion criteria, which may also have introduced selection bias. Additionally, as an observational study, our research identified associations but could not establish a causal relationship between coagulation marker alterations and DVT. Moreover, functional alterations in coagulation mechanisms may have been overlooked, as we only assessed the serum levels of the markers. Future research should expand on this by incorporating functional analysis to provide a more comprehensive understanding of the mechanisms involved.

## 5. Conclusions

Given the significant prothrombotic correlation that exists between proteins C and S, we propose that variations in their levels can serve as valuable markers in the evaluation of thrombotic risk during the final stages of pregnancy. In addition, despite their statistically significant higher values in the DVT group, due to the lack of FDP sensitivity and specificity, we believe that we need to evaluate additional markers to improve the detection of pregnant women at risk of developing DVT, such as the FDP/D-dimer ratio.

No statistically significant differences were observed for any of the other investigated markers (FDP, β2-GP1, APOH, and factor V) in terms of serum levels between women with a normal pregnancy and those who developed DVT, suggesting that the evaluation of these markers is not helpful in estimating the risk of DVT. An interesting aspect that needs further evaluation, especially due to scarce research on this topic, is the possible thrombotic-protective effect of higher factor V plasma levels.

## Figures and Tables

**Table 1:** The demographic characteristics and coagulation parameters of our included cases.

Variable	Group	Mean	St.Dev.	95.0% CI for Mean		Percentiles			Min	Max
				LL	UL	25%	Median 50%	75%		
Age (years)	control	27.21	5.08	25.65	28.77	23	28	30	18	37
	DVT	28.97	7.68	26.42	31.51	22	31	34	18	41
Gestational age (weeks)	control	36.63	3.06	35.69	37.57	35	37	38	28	42
	DVT	35.48	4.69	33.70	37.27	32	37	39	23	41
FDP (μg/mL)	control	0.79	0.16	0.74	0.84	0.70	0.79	0.93	0.47	1
	DVT	3.54	2.64	2.54	4.55	1.18	3.15	4.88	0.47	10
APL (ng/mL)	control	355.66	67.68	334.83	376.49	312.79	345.29	399.75	221.85	474
	DVT	251.47	112.08	208.84	294.10	183.88	235.51	355.97	87.45	474
Protein C (μg/mL)	control	10.46	2.38	9.73	11.20	8.99	10.44	11.84	5.85	14
	DVT	10.53	2.52	9.57	11.48	8.70	10.84	11.28	5.85	20
Protein S (μg/mL)	control	0.88	0.27	0.80	0.97	0.74	0.85	0.95	0.52	2
	DVT	0.40	0.45	0.23	0.58	0.09	0.10	0.87	0.01	2
APOH (μg/mL)	control	430.12	294.40	339.52	520.72	196.00	354.44	644.93	103.59	980
	DVT	356.81	343.00	226.34	487.28	124.21	229.91	465.00	31.55	1452
Human factor V (ng/mL)	control	9.11	5.10	7.54	10.68	5.71	8.36	11.61	2.29	34
	DVT	8.24	3.63	6.86	9.62	5.48	8.31	11.61	2.29	15

μg, micrograms; mL, milliliter; ng, nanograms; St.Dev., standard deviation; CI, confidence interval; LL, lower limit; and UL, upper limit (FDP-fibrinogen/fibrin degradation products; APL-antiphospfolipid; APOH-apolipoprotein H).

**Table 2:** Statistical significance of the demographic characteristics and coagulation parameters between the control group and the DVT group.

	Mann–Whitney U	Z	Asymp. Sig. (Two-Tailed)
Age (year)	500,500	−1.416	0.157
Gestational age (week)	607,000	−0.191	0.848
FDP (μg/mL)	188,500	−4.996	0.000
APL (ng/mL)	280,000	−3.945	0.000
Protein C (μg/mL)	615,000	−0.098	0.922
Protein S (μg/mL)	248,000	−4.313	0.000
APOH (μg/mL)	499,000	−1.430	0.153
Human factor V (ng/mL)	574,500	−0.563	0.573

## Data Availability

The data used to support the findings of this study are available upon request from the corresponding author.
